# SiGe/Si Multi-Quantum-Well Micro-Bolometer Array Design and Fabrication with Heterogeneous Integration

**DOI:** 10.3390/mi12121553

**Published:** 2021-12-13

**Authors:** Zhong Fang, Yong He, Zhequan Chen, Yunlei Shi, Junjie Jiao, Xuchao Pan

**Affiliations:** 1 School of Mechanical Engineering, Nanjing University of Science and Technology, Nanjing 210094, China; fangzhongah@163.com (Z.F.); jjj120@njust.edu.cn (J.J.); pxchxc@njust.edu.cn (X.P.); 2 School of Mechanical Engineering, Zhejiang Industry & Trade Vocational College, Wenzhou 325000, China; chenzhequan1993@163.com; 3 Quality Inspection and Testing Center, China Electronic Product Reliability and Environmental Testing Research Institute, Guangzhou 510610, China; 13770562272@163.com

**Keywords:** SiGe/Si MQWs, micro-bolometer, heterogeneous integration, adhesive bonding

## Abstract

The micro-bolometer is important in the field of infrared imaging, although improvements in its performance have been limited by traditional materials. SiGe/Si multi-quantum-well materials (SiGe/Si MQWs) are novelty thermal-sensitive materials with a significantly high TCR and a comparably low 1/f noise. The application of such high-performance monocrystalline films in a micro-bolometer has been limited by film integration technology. This paper reports a SiGe/Si MQWs micro-bolometer fabrication with heterogeneous integration. The integration with the SiGe/Si MQWs handle wafer and dummy read-out circuit wafer was achieved based on adhesive wafer bonding. The SiGe/Si MQWs infrared-absorption structure and thermal bridge were calculated and designed. The SiGe/Si MQWs wafer and a 320 × 240 micro-bolometer array of 40 µm pitch L-type pixels were fabricated. The test results for the average absorption efficiency were more than 90% at the wavelength of 8–14 µm. The test pixel was measured to have a thermal capacity of 1.043 × 10^−9^ J/K, a thermal conductivity of 1.645 × 10^−7^ W/K, and a thermal time constant of 7.25 ms. Furthermore, the total TCR value of the text pixel was measured as 2.91%/K with a bias voltage of 0.3 V. The SiGe/Si MQWs micro-bolometer can be widely applied in commercial fields, especially in early medical diagnosis and biological detection.

## 1. Introduction

Infrared (IR) imaging detectors have been widely used in military and commercial fields, such as threat detection, target recognition, surveillance, medical diagnosis and automotive night-vision [1]. Since the 2019-nCoV outbreak, the demand for infrared imaging detectors for the early detection of suspected cases has vastly increased [2]. 

In recent decades, uncooled thermal detectors, represented by micro-bolometers, have become the most important type in the field of infrared imaging. The performance of micro-bolometers has greatly improved, with this advance being driven primarily by the development of the micro-fabrication process [3]. One of the evaluation parameters for a high-performance micro-bolometer is the thermal coefficient resistance (TCR).

The SiGe/Si multi-quantum-well materials (SiGe/Si MQWs) is a new-generation thermal-sensitive 2D material based on energy band engineering [4]. This material consists of several monocrystalline SiGe/Si quantum-well layers and two thick stress-buffer layers that are used to prevent structural failure in a high-temperature environment during the epitaxial process. Each SiGe/Si quantum-well contains a p-doped monocrystalline Si layer as the barrier and a p-doped monocrystalline SiGe layer as the quantum well. Compared with the traditional materials applied in the micro-bolometer, it has a comparably high TCR (2.8–5.5%/K) by controlling the Ge content of the monocrystalline SiGe layer [5,6]. Moreover, this material has a low 1/f noise due to its monocrystalline structure [7]. 

However, the SiGe/Si multi-quantum-well materials, despite these advantages, face difficulties in the integration process, which limit their application in micro-bolometer fabrication. In the conventional micro-bolometer manufacturing process, a thermal coefficient resistance film is deposited on the CMOS read-out circuit wafer, and the thermal insulation micro-bridge structure is constructed by the micro-fabrication process. The SiGe/Si MQWs are based on alternating epitaxial-grown monocrystalline Si layers and monocrystalline SiGe layers, in which the process temperature is above 575 centigrade and the epitaxial-grown substrate must be monocrystalline Si. However, the CMOS read-out circuit wafers were tolerant of neither process temperatures above 475 centigrade nor monocrystalline epitaxy [8]. The application of such high-performance monocrystalline films in a micro-bolometer has been limited by film integration technology. 

In recent years, due to the development of 3D integration technology, silicon on integrated-circuit wafers can easily be fabricated by the wafer bonding of the material wafer and CMOS circuit wafer [9,10]. This technique has increased the variety of high-performance monocrystalline thin-film applications, facilitating the fabrication of complicated microstructures integrated with IC wafers. Through the 3D integration technology, the SiGe/Si multi-quantum-well material can be used in the fabrication of a high-performance micro-bolometer.

In this paper, we report a micro-bolometer array based on the SiGe/Si multi-quantum-well materials. The SiGe/Si MQWs infrared-absorption structure and thermal bridge were analyzed by the simulation, and the structure of the micro-bolometer was designed. Based on adhesive wafer bonding, a heterogeneous integration with the SiGe/Si MQWs handle wafer and dummy read-out circuit wafer was achieved. The SiGe/Si MQWs handle wafer and a 320 × 240 micro-bolometer array of 40 µm pitch L-type pixels were fabricated. A thermal response-measured system was built to evaluate the performance of the test pixels. Finally, the TCR, thermal capacity, thermal conductivity, and thermal time constant of the micro-bolometer pixel were tested by the measuring system.

## 2. Materials and Methods

### 2.1. The Energy Band Analysis of SiGe/Si MQWs and TCR Calculation

**Figure 1:**
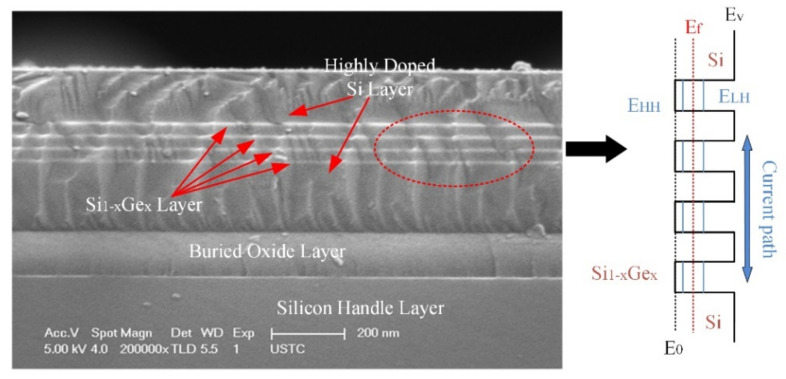
SEM photograph of the SiGe/Si MQWs with the 30% Ge content and diagram of its energy band.

The SiGe/Si multi-quantum-well materials consist of an alternating monocrystalline SiGe layer and an Si layer (as shown in Figure 1). As the bandgap of the SiGe layer is narrower than that of the Si layer, the valence band of these materials is discontinuous. The SiGe layer forms potential wells that will trap the holes, and these holes can only move perpendicularly to the SiGe layer plane. The holes in the SiGe potential wells become free carriers with thermal excitation. The concentration of free carriers is correlated with the temperature, so the SiGe/Si multi-quantum-well materials have a high-resistance temperature coefficient. The thermal coefficient resistance (*TCR*) of this material can be calculated by [11]:

(1) $$TCR(T)=-\frac{1}{kT^{2}}\left(\frac{3}{2}kT+E_{f}-E_{v}\right)$$

Here, *TCR (T)* is the thermal coefficient resistance of this material, *E_f_* is the Fermi level of this material, *E_v_* is the valence-band edge of this material, *k* is the Boltzmann constant, and *T* is the absolute temperature. The Ge content in the SiGe layer directly affects the value of *E_f_ − E_v_* and the TCR of this material. The value of *E_f_ − E_v_* can be calculated with the Schrodinger equation, Poisson equation, and current-continuity equation [12,13,14,15]. The theoretical calculation values with different Ge concentrations in the SiGe layer are listed in Table 1 (when the boron concentration in the SiGe layer and Si layer is 1 × 18 cm^−3^, the thickness of the SiGe layer is 10 nm, and the thickness of the Si layer is 30 nm). When the Ge concentration in the SiGe layer increases to more than 10%, the TCR value of the material greatly improved. 

However, a too high Ge content will lead to an increase in dislocations during the material-epitaxy process. The Ge content in the epitaxial growth process of the SiGe/Si MQWs needs to be optimized according to the process capability. 

### 2.2. The Infrared-Absorption Structure Design

**Figure 2:**
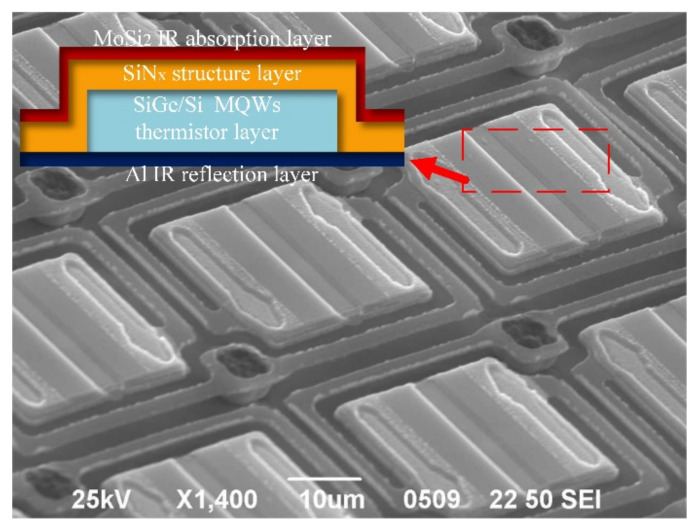
L-type SiGe/Si MQWs micro-bolometer and its infrared-absorption structure sketch.

As shown in Figure 2, the micro-bolometer pixel consists of four function-based layers: (a) the molybdenum silicide infrared-absorption film, (b) the SiGe/Si multi-quantum-well thermistor layer, (c) the silicon nitride structure layer, (d) the aluminum infrared reflection layer. These layers formed a Fabry–Perot resonant optical cavity to absorb the incident infrared radiation, resulting in a temperature change in the resonant optical cavity. These temperature changes were detected by the SiGe/Si multi-quantum-well thermistor, and the changes were converted into an electrical signal by the read-out circuits. Then, the image of the incident infrared radiation was obtained by processing the read-out electrical signal. The performance of the micro-bolometer was greatly affected by the infrared absorptivity of the resonant optical cavity, which was within the specified wavelength.

Based on the optical thin-film theory, an optical matrix for the multi-layer film system is defined (shown in Equation (2)) for the resonant optical cavity [16,17]

(2) $$\left[\begin{array}{c} B\\ C \end{array}\right]=\prod_{j=1}^{k}\left[\begin{array}{cc} \cos\delta_{j} & \eta_{j}{}^{-1}\sin\delta_{j}\\ i\eta_{j}\sin\delta_{j} & \cos\delta_{j} \end{array}\right]\left[\begin{array}{c} 1\\ \eta_{j+1} \end{array}\right],$$

where [*B C*] ^T^ is the characteristic matrix for the multi-layer film system, *k* is the number of layers in the system, *η_j_* is the optical admittance of film layer *j*, and *η*_*j*+1_ is the optical admittance of the next layer. Here, only the normal incidence of infrared radiation is considered. Moreover, the phase thickness *δ_j_* of layer *j* can be calculated by:

(3) $$\delta_{j}=\frac{2\pi }{\lambda }\eta_{j}d_{j}.$$

Here, *λ* is the wavelength of the incident radiation, *d_j_* is the film thickness of layer *j*. The p-like and s-like of the film’s optical admittance *η_j_* is defined as

(4) $$\eta_{j-p}=n_{j}/\cos\theta_{j},$$

(5) $$\eta_{j-s}=n_{j}\times \cos\theta_{j}.$$

where *n_j_* is the complex refractive index of layer *j*; *θ_j_* is the infrared radiation incident angle of layer *j*. The transmissivity *T* and reflectivity *R* of the multi-layer film system can be derived as:

(6) $$T=\frac{4\eta_{0}\eta_{j+1}}{(\eta_{0}B+C){\left(\eta_{0}B+C\right)}^{*}},$$

(7) $$T=\frac{4\eta_{0}\eta_{j+1}}{(\eta_{0}B+C){\left(\eta_{0}B+C\right)}^{*}}.$$

Here, *η*_0_ is the optical admittance of the vacuum. In summary, the absorption ratio *A* of the resonant optical cavity can be calculated as follows:

(8) A=1−T−R.

In the application, the infrared radiation in the atmosphere has a high-transmittance window at the wavelength of 8–14 µm. To achieve a better performance, the resonant optical cavity is optimized to obtain a high absorption efficiency within the working wavelength range.

**Figure 3:**
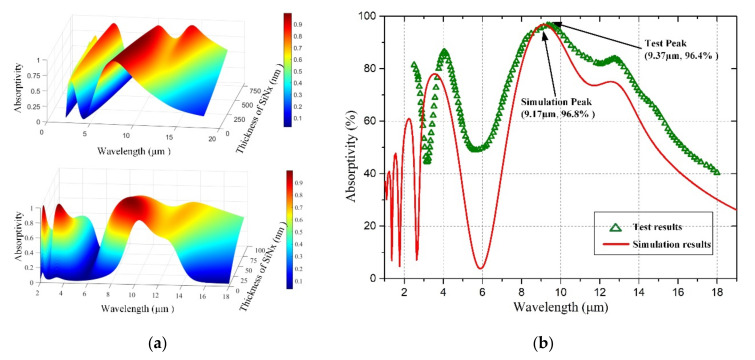
(**a**) The infrared-absorption surface plot of the resonant optical cavity; (**b**) The infrared-absorption simulation results and test results of the resonant optical cavity test structure.

According to the film’s optical parameters, applied in Table 2, the infrared-absorption surface plot of the resonant optical cavity was obtained by the MATLAB simulation [18,19]. The dependence of the resonant optical cavity on the normal incident infrared wavelength and critical layer thickness is shown in Figure 3a. Considering the fabrication error, the infrared-absorption structure is designed with an average absorption efficiency of 90%, in a wavelength of 8–14 µm. The optimized thickness range of the MoSi_2_ infrared-absorption layer is 15–35 nm; the optimized thickness range of the SiN_x_ structure layer is 300–400 nm. The test structure of the resonant optical cavity is fabricated to test the infrared absorptivity of the micro-bolometer. The respective layer thickness of the test structure is shown in Table 2.

The infrared-absorption simulation of the test structure is shown as the solid red line in Figure 3b. The test result of the infrared absorption is shown as triangular datapoints in Figure 3b. The average absorption efficiency of the test structure achieves more than 90% at the wavelength of 8–14 µm. The measured result is consistent with the calculated result, and the measured result has a higher average absorption efficiency within the working wavelength range.

### 2.3. The Bolometer Pixel Thermal Bridge Analysis

The micro-bolometer pixel absorbs the infrared radiation, and the infrared-absorption structure is heated by these incident infrared radiations. The temperature will rise following the infrared-absorption structure of the micro-bolometer pixel and is finally transmitted to the substrate through the cantilever and Au pillar. The performance of the micro-bolometer is greatly affected by the parameters of the pixel thermal bridge.

The temperature field changes in a micro-bolometer pixel can be characterized by the heat balance Equation (9) [20]:

(9) $$C\frac{dT}{dt}=Q-G\left(T-T_{s}\right).$$

Here, *T_s_* is the initial temperature (substrate temperature), *Q* is the heat input, *C* is the thermal capacity of the pixel, and *G* is the thermal conductivity of the pixel.

The thermal conductivity *G* of the pixel can be derived as

(10) $$G=G_{leg}+G_{radiation}+G_{gas}+G_{convection}.$$

where *G_leg_* is the thermal conductivity of the cantilever, *G_radiation_* is the thermal conductivity caused by thermal radiation, *G_gas_* is the thermal conductivity of the atmosphere, and *G_convection_* is the thermal conductivity caused by thermal convection. *G_gas_* can be calculated by [21]:

(11) $$G_{gas}=\beta_{pixel}A_{pixel}/d\left[\lambda_{gas}{}^{-1}+{\left(\gamma_{gas}pd\right)}^{-1}\right].$$

Here, *β_pixel_* is the fill factor of the micro-bolometer, *A_pixel_* is the micro-bolometer pixel area (pixel pitch), *p* is the gas pressure, *d* is the distance between the infrared-absorption structure and the substrate (about 3 μm), *λ_gas_* is the pressure-independent thermal conductivity of the gas in the high-pressure regime (*λ_a_* = 0.024 W/K·m for standard atmospheric pressure), and *γ_gas_* is the thermal conductivity per unit pressure and length in the low-pressure regime (*γ_a_* = 1.9 m/K·s for standard atmospheric pressure). Under vacuum and room temperature, the thermal conductivity of cantilever *G_leg_* is several orders of magnitude greater than other thermal conductivities (*G_gas_* + *G_convection_* + *G_convection_*) of a micro-bolometer pixel. The thermal conductivity of cantilever *G_leg_* can be calculated by

(12) $$G_{leg}=\sum \lambda_{leg-m}A_{leg-m}/l_{leg}.$$

where *λ_leg-m_* is the thermal conductivity coefficient of the cantilever material, *A_leg-m_* is the cross-section area of the cantilever, and *l_leg_* is the length of the cantilever. The thermal conductivity of the micro-bolometer pixel calculation is shown in Table 3. The total thermal conductivity of the micro-bolometer pixel is approximately 1.64 × 10^−7^ W/K.

The thermal capacity *C_p_* of the pixel can be defined as:

(13) $$C_{p}=V_{1}\rho_{1}c_{1}+V_{2}\rho_{2}c_{2}+\ldots \ldots ..$$

Here, *V_x_* is the volume of layer x, *ρ_x_* is the density of layer x, and *c_x_* is the specific thermal capacity of layer x. The micro-bolometer pixel thermal capacity calculation is shown in Table 4. The total thermal capacity of the micro-bolometer pixel is approximately 1.2574 × 10^−19^ J/K.

The thermal response time of the micro-bolometer pixel is determined by the thermal capacity of the pixel and the thermal conductivity between the infrared-absorption structure and substrate. The thermal time constant *τ_p_* of the pixel can be calculated by [21]:

(14) $$\tau_{p}=C_{p}/G.$$

With the thermal capacity of 1.2574 × 10^−9^ J/K and thermal conductivity of 1.64 × 10^−7^ W/K, the thermal time constant *τ_p_* of the micro-bolometer pixel is 7.67 ms.

## 3. Results

### 3.1. Adhesive Wafer Bonding and Micro-Bolometer Fabrication

**Figure 4:**
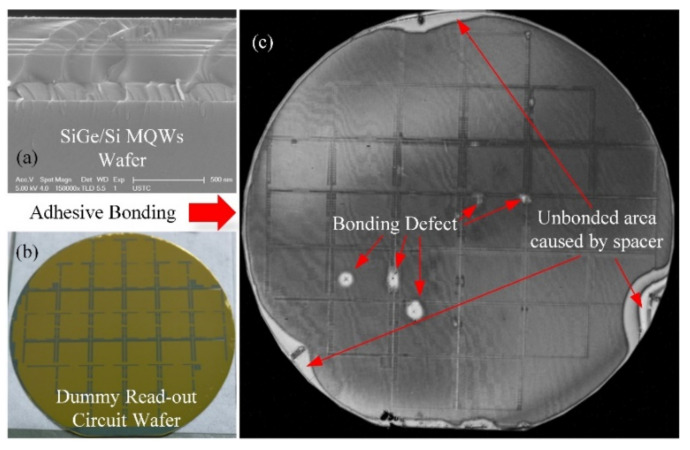
Sketch of the heterogeneous integration between SiGe/Si MQWs wafer and read-out wafer: (**a**) The cross-section of SiGe/Si MQWs wafer; (**b**) The overview of dummy read-out circuit wafer; (**c**) The ultrasonic micrograph of bonded wafers.

A 100 mm SOI wafer was used as the handle wafer for the SiGe/Si MQWs epitaxial growth. The buried layer of this wafer was 1.5 µm ±10% and the SOI layer of this wafer was 180 nm ± 5%. The SiGe/Si MQWs were deposited in the reduced-pressure chemical vapor deposition (RPCVD, Epsilon 2000, ASM, Versterkerstraat, The Netherlands) reactor with the process condition of pressure 2600 Pa and a temperature of 650 centigrade. A total thickness of 370 nm monocrystalline membrane was deposited on the SOI layer (shown in Figure 4a). Considering the lattice dislocation during the epitaxial process, the Ge concentration of the SiGe layer in SiGe/Si MQWs was 30%. A 20 nm-thick Ti and 75 nm-thick Al were sputtered (LAB18, Kurt J. Lesker, Jefferson Hills, PA, USA) on top of the SiGe/Si MQWs to define the micro-bolometer pixel backside reflection layer.

The dummy read-out circuit wafer used a 100 mm Si wafer (100) as the substrate. The Au lines and Au pads of the read-out wafer were fabricated by magnetron sputtering and the lift-off process. These were used to electrically address the test pixel in the micro-bolometer arrays for characterization purposes and provided a current path during the electroplating of Au via pillars (shown in Figure 4b). The read-out wafer had a surface topography of about 300 nm, which is similar to most commercial CMOS wafers. The dummy read-out was used to replace the CMOS read-out IC wafer for fabrication experiments.

Both the SiGe/Si MQWs wafer and read-out wafer were spin-coated at 1500 rpm for 30 s with the adhesive-bonding polymer layers (mr-I 9100 m, Micro-resist Technology, BerlinGER). After that, the wafers were soft-baked at 100 centigrade for 15 min to remove the solvent and polymer pre-curing. The thickness of each adhesive-bonding polymer layer was about 1400 nm, and the total thickness of the polymer layer was about 2800 nm. The bonding defects caused by particles and topography were reduced at this thickness. Then, the wafers were heterogeneously integrated using back-alignment adhesive wafer bonding (BA6/CB-6L, SUSS Micro-Tec, Garching, GER). Figure 4c was the ultrasonic micrograph of the heterogeneous integration wafer. Several bonding defects caused by particles and three unbonded areas caused by three bonder spacers were found. The test was operated in a public laboratory, and the heterogeneous integration yield will have a higher estimate in Fab.

**Figure 5:**
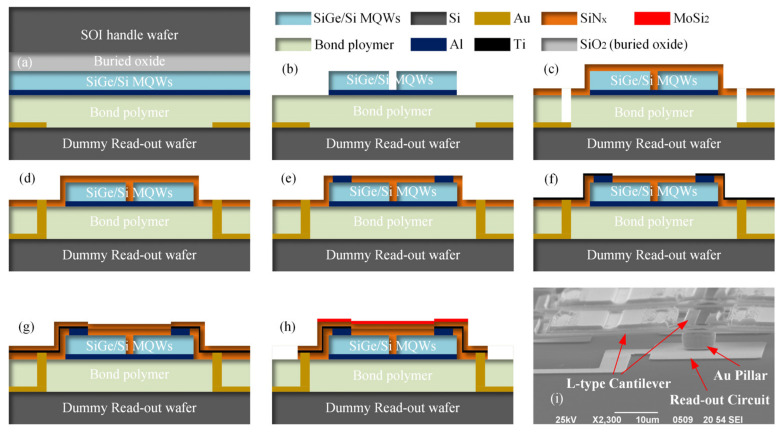
Micro-bolometer fabrication process: (**a**) The heterogeneous integration; (**b**) The SiGe/Si MQWs thermistor defined; (**c**) The SiN_x_ layer deposited and the etched via-holes; (**d**) The Au pillar electroplate; (**e**) The Al contact pads that were defined; (**f**) The patterned Ti connect layer; (**g**) The deposited SiN_x_ layer; (**h**) The deposited MoSi_2_ layer and the defined pixel; (**i**) The final pixel.

The Si handle layer of the SiGe/Si MQWs wafer (shown in Figure 5a) was removed by the SF_6_-based inductively coupled plasma (ICP) etching (MPX HRM System, STS, Wells, UK and self-stopped at the buried oxide layer. The buried oxide was removed by the buffered HF (10:1) and stopped at the SOI layer. As shown in Figure 5b, the SiGe/Si MQWs layer was patterned by lithography (MA6/BA6, SUSS Micro-Tec, Garching, GER) and CF_4_-based reaction ion etch (RIE, Tegal 903e, Tegal, Petaluma, CA, USA), while the Al reflection layer was etched by Ar-based ion beam etch (IBE, IBE-A-150, BCT, Beijing, PRC). Then, the first SiN_x_ layer, 200 nm thick, was deposited by plasma-enhanced vapor deposition (PECVD, Plasmalab System 100, OxFord Instrument, Oxford, UK) with the process conditions of a pressure of 230 Pa and temperature of 200 degrees centigrade. 

Figure 5c shows that the via-holes through the SiN_x_ layer and polymer layer were etched by RIE (Plasmalab System 80, OxFord Instrument, Oxford, UK). The Au pillars were constructed by electroplating to fill the via-holes (shown in Figure 5d). Then, the Al contact pads were defined with lithography and lift-off processes (shown in Figure 5e). A 50 nm-thick Ti layer was deposited and patterned by magnetron sputtering and IBE etching. The second SiN_x_ layer of 175 nm and the MoSi_2_ layer of about 20 nm were deposited, as shown in Figure 5g,h). After that, the cantilevers and the infrared-absorption structure were defined by lithography and the dry-etching process (shown in Figure 5h). Finally, the polymer layer was completely removed by isotropic oxygen plasma etching (shown in Figure 5i), and the contact resistances between the Si and Al layers were decreased by annealing in an Ar atmosphere.

### 3.2. Structural Characterization of Micro-Bolometer Array

**Figure 6:**
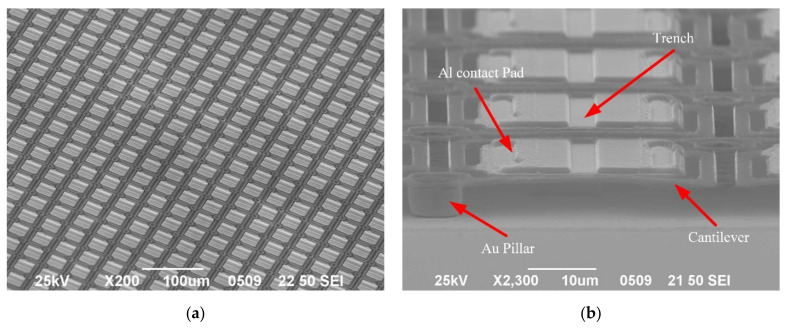
(**a**) The SEM graph of the partial micro-bolometer array; (**b**) The SEM graph of several L-type pixels of the micro-bolometer array.

The structures after the fabrication processes are indicated in Figure 6. Each micro-bolometer die consisted of several test pixels and 320 × 240 pixels of a 40 × 40 µm pitch (shown in Figure 6a). A pixel constituted two Au pillars, two L-type cantilevers, and one infrared-absorption structure (shown in Figure 6a). The L-type cantilevers supported the pixel freestanding on the substrate, while the thermal infrared-absorption structure only supported conductive coupling to the substrate through the cantilevers and Au pillars in a vacuum. The electrical path of the pixel was connected to the Au pillars, while Ti connected the layer of the cantilevers, Al contact pads, SiGe/Si MQWs thermistor, and the Al reflection layer. The electrical connection structure of the pixel was intact. A 100 mm wafer had 30 micro-bolometer dies. Dies with a pixel-integrity ratio of more than 95% accounted for more than 70% of the total through the structural characterizations and statistics.

### 3.3. Electrical Characterization of Micro-Bolometer Pixel

**Figure 7:**
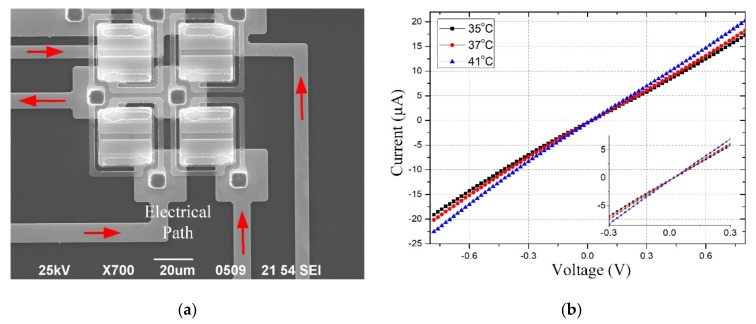
(**a**) The electrical path of test pixel; (**b**) The I–V curves of test pixels with different temperatures.

The micro-bolometer die was packaged in a metal shell. Au wires connected the die electrodes and the pads of the shell. Each micro-bolometer die had several test pixels, where the die electrodes and the Au pillars of the test pixels connected via a read-out circuit. The electrical path of the test pixels is shown in Figure 7a. The I-V curves with different temperatures are shown in Figure 7b. The TCR of the test pixels can be calculated as [22]:

(15) $$TCR=\frac{2\times \left(R_{2}-R_{1}\right)}{\left(R_{2}+R_{1}\right)\times \left(T_{2}-T_{1}\right)}.$$

where *R*_1_ is the resistance of test pixels at temperature *T*_1_, and *R*_2_ is the resistance of test pixels at temperature *T*_2_.

The average TCR of pixels was approximately 2.62%/K, with an applied voltage ±0.8 V. When the applied voltage decreased to ±0.3 V, the average TCR of pixels was increased to about 2.91%/K. 

**Figure 8:**
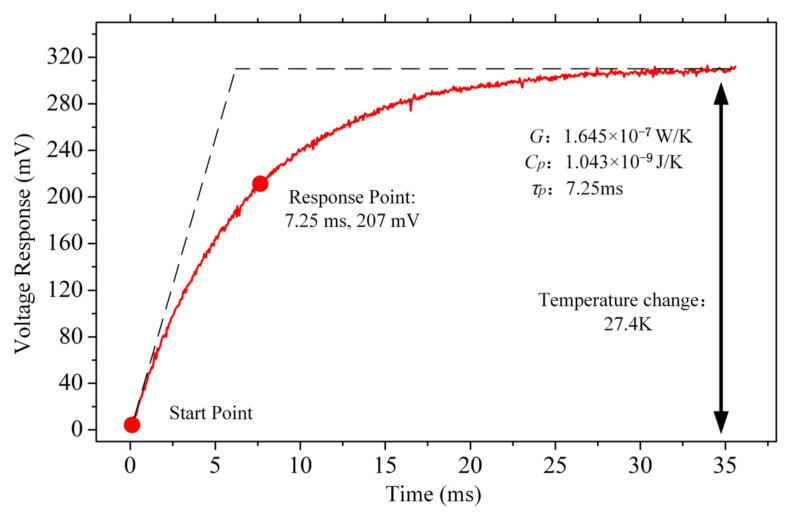
The test pixel thermal response curve of a micro-bolometer array.

A thermal response-measured system was built to evaluate the performance of the test pixels. The measuring system was formed by a vacuum pump, Wheatstone bridge, and data acquisition board (NIUSB-6366, National Instruments, Austin, TA, USA). During the test, the vacuum pump was used to ensure that the pressure in the metal shell was less than 1.3 Pa. Then, the bias voltage of 0.3 V square pulses was applied to the Wheatstone bridge, and the data acquisition board collected the responding voltage data (shown in Figure 8). According to Equation (9), the relation between the thermal time constant *τ_p_* and thermal steady-state time *τ*_0_ can be derived as [22]:

(16) $$\frac{\tau_{p}}{\tau_{0}}=\frac{1-e^{-1}}{1}\approx 0.63.$$

As shown in Figure 8, the thermal time constant of the test pixel is 7.25 ms. The thermal conductivity *G* of the test pixel can be calculated by [22,23]

(17) $$G=I\times V_{0}\times TCR\times \frac{R_{s}}{R_{0}}.$$

Where *I* is the bias current, *V*_0_ is the thermal steady-state voltage, *R_s_* is the resistance of the pixel at the start point, and *R*_0_ is the resistance of the pixel at the thermal steady state. The thermal conductivity of the test pixel was 1.645 × 10^−7^ W/K, according to calculations. According to Equation (13), the thermal capacity of the test pixel was 1.043 × 10^−9^ J/K.

## 4. Discussion and Conclusions

This paper presents a SiGe/Si MQWs micro-bolometer array designed and fabricated based on heterogeneous integration. The SiGe/Si MQWs materials were analyzed and deposited. The infrared-absorption structure and the thermal bridge of the micro-bolometer were calculated and designed. The infrared-absorption structure was designed with an average absorption efficiency of 90% in the wavelength range of 8–14 µm. The micro-bolometer pixel was designed with a thermal capacity of 1.2574 × 10^−9^ J/K, a thermal conductivity of 1.64 × 10^−7^ W/K, and a thermal time constant of 7.67 ms.

Based on adhesive wafer bonding, a heterogeneous integration with the SiGe/Si MQWs handle wafer, and the dummy read-out circuit wafer was achieved. A 320 × 240 micro-bolometer array of 40 µm pitch L-type pixels was fabricated. The test pixel was measured with a thermal capacity of 1.043 × 10^−9^ J/K, a thermal conductivity of 1.645 × 10^−7^ W/K, and a thermal time constant of 7.25 ms. The infrared absorption of the micro-bolometer was measured with an average absorption efficiency of 90% in the wavelength range of 8–14 µm. This proves the effectiveness of the micro-bolometer structural design. The total TCR value of the text pixel was measured as 2.91%/K, with a bias voltage of 0.3 V, which is significantly higher than traditional materials. The SiGe/Si MQWs micro-bolometer can be widely applied in commercial fields, especially in early medical diagnosis and biological detection, with continuous improvements in its performance and fabrication efficiency.

## Figures and Tables

**Table 1:** The TCR calculation results of the SiGe/Si MQWs with different Ge concentrations.

Ge Content	10%	20%	25%	30%	35%	50%
*E_f_ −E_v_* (eV)	0.099	0.152	0.182	0.214	0.248	0.358
*TCR* (%/K)	1.78	2.47	2.85	3.26	3.69	5.12

**Table 2:** The film optical parameters of the infrared-absorption structure.

	Refractive Index *n*	Extinction Coefficient *κ*	Thickness (nm)
MoSi_2_ ^1^	4.7	5.3	21
SiN_x_	2	*κ_SiN_* ^2^	375
Si ^3^	3.4	0	510
Si_0.7_Ge_0.3_ ^3^	3.58	0	40
Al	25	68	75

^1^ The refractive index and extinction coefficient of MoSi_2_ is measured by Infrared Ellipsometer (M-2000DI, J.A. Woollam, Lincoln, Dearborn, MI, USA). ^2^ The doping level of the SiGe/Si MQWs is 1 × 10^18^ cm^−3^. ^3^ The extinction coefficient of SiN_x_ is defined as: *κ_SiN_* = 0.1 + 1.76exp {−0.5[(λ − 11.45)/1.25]^2^}.

**Table 3:** The calculation of the micro-bolometer pixel thermal conductivity.

	*λ_leg-m_* (W/(m·K))	*A_leg-m_* (μm^2^)	*l_leg_* (μm)	*G_leg_* (10^−8^ W/K)
SiN_x_	3.2	2 × 0.75	56	8.58
Ti	21.9	2 × 0.1	56	7.82
Total				16.40

**Table 4:** The calculation of the micro-bolometer pixel thermal conductivity.

	Thickness (nm)	*V_x_* (μm^3^)	*ρ_x_* (kg/m^3^)	*c_x_* (W/(m·K))	Capacity (10^−9^ J/K)
SiN_x_	375	270.7	3440	710	0.6612
Si	180	78.41	2329	713	0.1302
Doped Si	140	79.7	2329	713	0.1323
Si_0.7_Ge_0.3_	40	22.8	3331	578.6	0.0439
Doped Si	190	108.1	2329	713	0.1795
Al	75	46.4	2702	880	0.1103
Total					1.2574
